# Architectural model for muscle growth during maturation

**DOI:** 10.1007/s10237-021-01492-y

**Published:** 2021-07-24

**Authors:** Stefan Papenkort, Markus Böl, Tobias Siebert

**Affiliations:** 1 Department of Motion and Exercise Science, University of Stuttgart, Stuttgart, Germany; 2 Institute of Mechanics and Adaptronics, Technische Universität Braunschweig, Braunschweig, Germany

**Keywords:** Muscle model, Muscle architecture, Fascicle length, Pennation angle, Aponeurosis, Muscle morphology

## Abstract

Muscle architecture, which includes parameters like fascicle length, pennation angle, and physiological cross-sectional area, strongly influences skeletal muscles' mechanical properties. During maturation, the muscle architecture has to adapt to a growing organism. This study aimed to develop an architectural model capable of predicting the complete 3D fascicle architecture for primarily unipennate muscles of an arbitrary age, based on fascicle data for an initial age. For model development, we collected novel data on 3D muscle architecture of the rabbit (*Oryctolagus cuniculus*) *M. plantaris* of eight animals ranging in age from 29 to 106 days. Experimental results show that *plantaris* muscle belly length increases by 73%, whereas mean fascicle length and mean pennation angle increases by 39 and 14%, respectively. Those changes were incorporated into the model. In addition to the data collected for *M. plantaris* the predictions of the model were compared to existing literature data of rabbit *M. soleus* and *M. gastrocnemius medialis*. With an error of −1.0 ± 8.6% for relative differences in aponeurosis length, aponeurosis width, muscle height, and muscle mass, the model delivered good results matching interindividual differences. For future studies, the model could be utilized to generate realistic architectural data sets for simulation studies.

## Introduction

The muscle’s architecture strongly influences the skeletal muscle’s mechanical properties (Gans and Gaunt 1991; Wickiewicz et al. 1984; Woittiez et al. 1983). It is commonly characterized by parameters like the fascicle length, the physiological cross-sectional area (PCSA), the angle of pennation, and aponeuroses’ dimensions (Kupczik et al. 2015; Papenkort et al. 2020). Moreover, the free tendon length (if present) is often considered when the entire muscle-tendon-complex’s (MTC) mechanical behavior is of interest.

In general, muscles with comparatively long fascicles show higher contraction velocities due to many sarcomeres in series. In contrast, comparatively short fascicles result in muscles with lower contraction velocity. However, when combined with a larger PCSA, they show an increased force production due to more parallel myofibrils (Lieber et al. 2000). On the MTC level, the ratio between muscle belly length and free tendon length plays an essential role in the system’s compliance and energetics (Mörl et al. 2016). MTCs with long tendons are, for example, able to store and recover large amounts of elastic energy during periodic movements.

During maturation, the increase in bone size requires adjustments in muscle length. Additionally, increases in force production and thus PCSA are necessary to compensate for increasing body weight during growth. As a result, the muscle has to undergo architectural changes to remain functional. Many studies dealt with architectural investigations of human and animal skeletal muscle (Bénard et al. 2011; Binzoni et al. 2001; Lieber and Blevins 1989). However, investigations on adaptations in muscle architecture during growth are sparse. Among studies on rabbits, Böl et al. (2016) determined the aponeuroses dimensions and fascicle length of *M. soleus* (SOL), *M. plantaris* (PLA), and *M. extensor digitorum longus* during growth. They found that increases in aponeuroses dimensions far exceeded the increase in fascicle lengths. In another study, Siebert et al. (2017) recorded the fascicle architecture of rabbit *M. gastrocnemius medialis* (GM) and *M. gastrocnemius lateralis*, *M. tibialis anterior*, and *M. flexor digitorum longus* for different stages of maturation. Their results showed similar findings, where substantial increases in aponeurosis length accompanied increases in muscle belly length. Fascicle lengths increased as well, whereas pennation angles remained nearly unchanged. Furthermore, Papenkort et al. (2020) carried out a detailed investigation of rabbit SOL architecture. Their results could support Böl et al.’s (2016) findings and showed indications for a homogeneous growth characteristic, where fascicle length distribution was preserved during maturation.

Different modeling attempts have been made to better understand and predict muscle growth. Various models deal with muscle architecture changes during active contraction (Randhawa and Wakeling 2015) with a varying degree of abstraction. Here, muscles are often reduced to line segments (Zajac 1989) or two-dimensional objects (Van Leeuwen 1992). Furthermore, muscle architecture is usually assumed to be homogeneous. To incorporate variation in architecture, Schenk et al. (2020) introduced a geometrical model based on 3D fascicle data that predicts the muscle shape and architecture for different muscle lengths. However, to the best of the authors’ knowledge, no model deals with 3D muscle architecture changes during growth. As mentioned before, increases in fascicle and aponeurosis length differ strongly during maturation, suggesting that muscle growth, e.g., cannot be captured by simple scaling. However, a realistic representation of the muscle’s architecture is important to predict its function (Woittiez et al. 1983; Wickiewicz et al. 1984; Gans and Gaunt 1991).

Therefore, this study presents a versatile 3D geometrical model that predicts different size muscles' architecture during maturation using fascicle coordinates from an initial data set. To provide model input, we collected new architectural data for the *M. plantaris*, spanning several months of age in rabbits. The study is structured as follows: The methods section starts with a description of the experimental data collection and includes definitions for considered architectural parameters considered. Another subsection introduces the architectural model. For model development, we used the entire experimental data of the *M. plantaris*. The results section starts with an analysis of the experimental data of the *M. plantaris* and continues with an analysis of model predictions for the *M. plantaris*, as well as the *M. soleus* (Papenkort et al. 2020) and *M. gastrocnemius medialis* (Siebert et al. 2017).

## Methods

### Preparation of the animals

This study was exempted from ethical committee review according to national regulations (German Animal Welfare Act), as we obtained rabbits (*Oryctolagus cuniculus*) from a slaughterhouse immediately after animal sacrifice. During their life, the rabbits had free access to food and grew up in bigger cells allowing for almost natural movement patterns. We recorded the muscle architecture as 3D fascicle coordinates of the PLA of eight animals (R1 to R8) ranging in age from 29 to 106 days. For more information about the animals' age and weight, see Table 3.

Preparations began by removing the left hind limbs' skin and thigh muscles, where the entire calf and foot section of the leg remained intact. We mounted the legs onto a frame to control ankle joint angles (65°) and knee joint angles (90°). Therefore, we inserted bone screws into the femur, distal tibia, middle foot, and front foot. Afterward, we stored the frames in Bouin’s solution (Böl et al. 2013), a fixative, for at least 7 d. The preparation process from slaughter to fixation took less than 3.5 h, during which the legs were cooled down and kept wet by applying Ca^++^-free Krebs solution. Finally, the legs were molded in wax. Therefore, standard candle wax was melted and subsequently cooled down close to its solidification point at around 40 °C in order to avoid heat damage to the tissue. The legs were placed upon the wax's surface and gently pushed into it, immersing them by approximately one half. After solidification, preparation ended by securely clamping the block of wax on a measuring table.

### Data acquisition and processing

For the muscle architecture acquisition, muscle fascicles were individually recorded with a Microscribe MLX digitizer with an internal accuracy of 0.076 mm. We went about this process by sequentially removing individual fascicles from the muscle belly with a tweezer, each leaving behind a small groove. While moving the measuring tip of the Microscribe MLX along this groove, coordinates were recorded at a frequency of 5 Hz, leading to a detailed geometric representation of the fascicle. Additionally, the muscle-tendon-complex's endpoints at the femur and Achilles tendon were recorded (raw data are available in the Online Resource). This methodology was validated in previous studies (Schenk et al. 2020; Wick et al. 2018; Papenkort et al.^2020^).

**Fig. 1:**
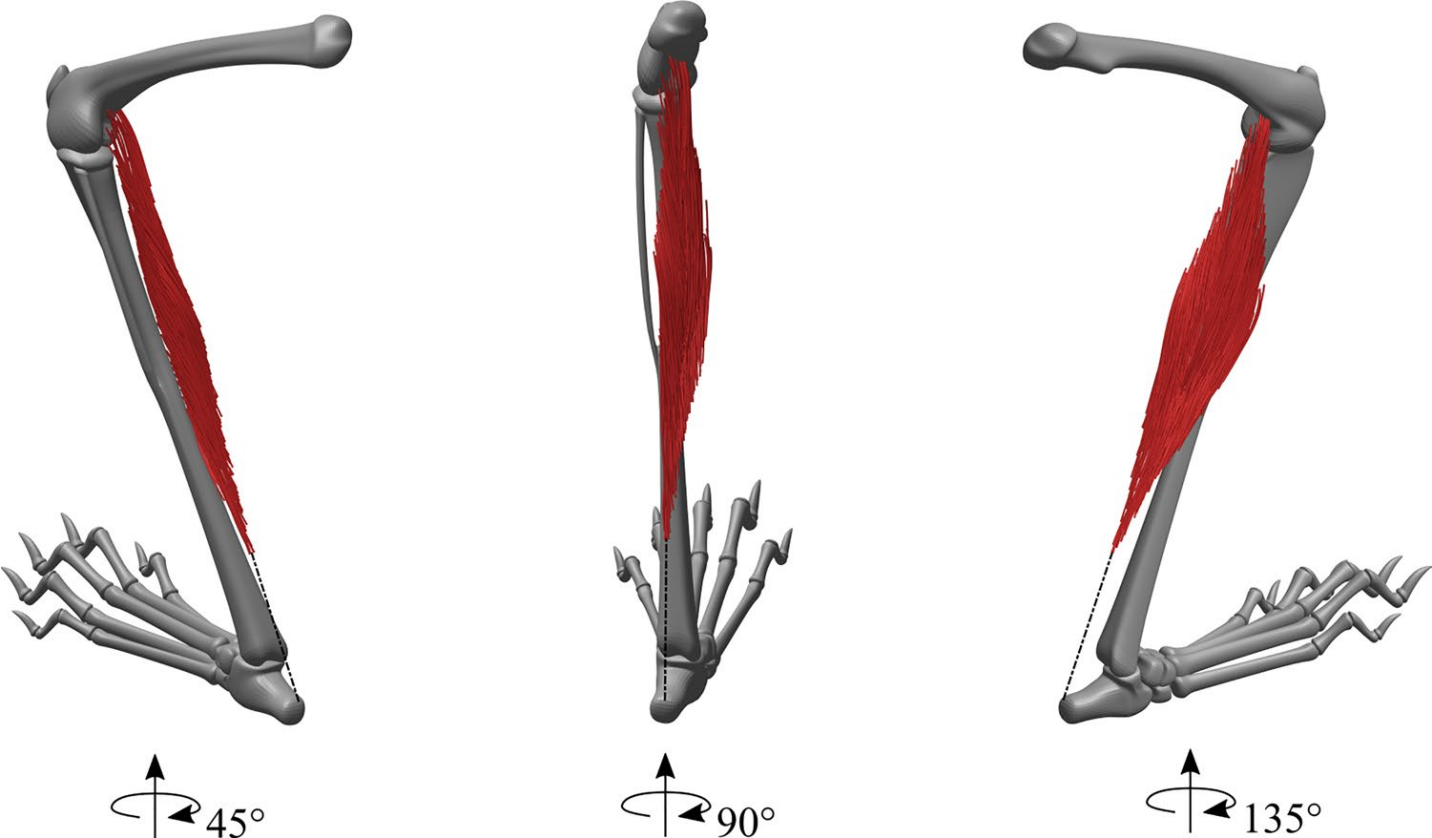
Visualization of the PLA of rabbit R6. The bone geometry was obtained from a standard CAD model of a rabbit skeleton that was scaled and adjusted in joint angles to fit the tracking data

The digitization process' result is a set of fascicle coordinates for several hundred fascicles, which were further processed with MATLAB (MATLAB R2018b, The MathWorks, Inc., Natick, MA, USA). Fascicle coordinates were first smoothed by quadratic fitting, as was previously done by Papenkort et al. (2020) (cf. Fig. 1). We subsequently evaluated the polynomials equidistantly to obtain 20 points along each fascicle's spatial path.

**Fig. 2:**
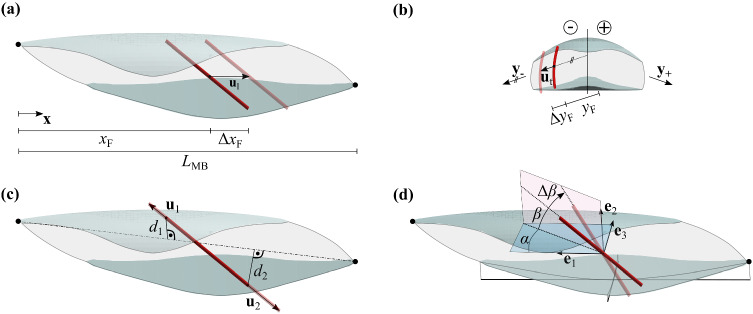
**a** Scaling in muscle belly length. The fascicle’s length displacement ${{\varvec{u}}}_{l}$ is parallel to the muscle’s length axis ${\varvec{x}}$. Its magnitude ${\Delta x}_{F}$ depends on its location ${x}_{F}$ in relation to the muscle belly length ${L}_{MB}$. **b** Scaling in muscle belly width. The fascicle’s transversal displacement ${{\varvec{u}}}_{t}$ is parallel to the transversal direction ${{\varvec{y}}}_{-}$ or ${{\varvec{y}}}_{+}$, depending on the side of the muscle belly cross section. Its magnitude ${\Delta y}_{F}$ depends on the distance ${y}_{F}$, which is scaled according to Eq. (3). **c** Fascicle lengthening. Here, fascicle endpoint displacement ${{\varvec{u}}}_{1}$ and ${{\varvec{u}}}_{2}$ depends on the distance of the respective endpoint (${d}_{1}$ or ${d}_{2}$) to the muscle’s line of action (dashed line), according to Eq. (4). **d** Fascicle rotation. For changes in pennation angle between the fascicle and the aponeurosis plane $\beta$ is changed by $\Delta \beta$. The transversal angle $\alpha$ remains unchanged

The fascicle’s length was computed as the sum of line segment lengths connecting the fitted fascicle points. For further definitions, we introduce a coordinates system (${\mathbf{e}}_{1},{\mathbf{e}}_{2},{\mathbf{e}}_{3}$) (cf. Fig. 2d), obtained by principal component analysis of fascicle endpoints. Here, ${\mathbf{e}}_{1}$ and ${\mathbf{e}}_{3}$ point in aponeurosis length and width direction, respectively, and together form the aponeurosis plane. They represent the average direction of the principal components for the individual aponeuroses shown in Fig. 7a.

For this coordinate system, $\alpha$ is the transversal deviation of the fascicle’s projection from the aponeurosis length axis ${\mathbf{e}}_{1}$, and the pennation angle $\beta$ is the vertical deviation from the aponeurosis plane. Furthermore, the aponeurosis length and width were defined for each of the two superficial aponeuroses as the largest distance of two aponeurosis points in ${\mathbf{e}}_{1}$ and ${\mathbf{e}}_{3}$ direction, respectively. The third principal component ${\mathbf{e}}_{2}$ points in the normal direction of the aponeurosis plane. The distance of the two aponeurosis center-points with respect to ${\mathbf{e}}_{2}$ was defined as the muscle's height. The muscle belly length was obtained by the same technique and followed to the largest distance of two fascicle points in ${\mathbf{e}}_{1}$ direction. The free tendon length of the Achilles tendon's PLA compartment was derived by subtracting the muscle belly length from the recorded MTC length. For an estimation of the muscle’s volume ${V}_{\mathrm{m}}$, we performed a boundary analysis on the entirety of tracked points by applying the MATLAB function *boundary* with a shrink factor of 0.6. The appropriate shrink factor ensured a tight fit around the point cloud that also included non-convex sections. The muscle mass resulted from multiplying the muscle’s volume with the skeletal muscle density of 1.056 g/cm^−3^ (Méndez 1960). Finally, we calculated the PCSA as

1 $$PCSA = \frac{{V}_{\mathrm{m}}}{{\tilde{L }}_{\mathrm{F}}},$$

where ${\tilde{L }}_{\mathrm{F}}$ is the mean fascicle length. Note that here fascicles are not necessarily at optimal length, implying that PCSA at optimal length may be different from what we compute here.

### Architectural model

For the presented architectural model, the assumption is made that muscle growth can be described by the superposition of four distinct geometrical operations, namely scaling in the length direction, scaling in the width direction, fascicle lengthening, and fascicle rotation. The model was developed based on a predominantly unipennate muscle with two large, superficial aponeuroses, like the rabbit PLA.

Scaling in the length direction is achieved by shifting individual fascicles in the aponeurosis’ length direction $\mathbf{x}$, where the magnitude of the displacement vector $\Delta {x}_{\mathrm{F}}$ can be expressed as

2 $$\Delta {x}_{\mathrm{F}}={\mathrm{x}}_{\mathrm{F}}\cdot \frac{{\Delta L}_{\mathrm{MB}}}{{L}_{\mathrm{MB}}}\cdot {f}_{\mathrm{c}}$$

(cf. Fig. 2a). Here, ${L}_{\mathrm{MB}}$ is the muscle belly length and ${\Delta L}_{\mathrm{MB}}$ is the change in muscle belly length. ${x}_{\mathrm{F}}$ represents the x-coordinate of the fascicle’s midpoint. ${f}_{\mathrm{c}}>1$ is a correction term ensuring that ${L}_{\mathrm{MB}}$ is actually increasing by $\Delta {L}_{\mathrm{MB}}$, since scaling is applied to fascicle midpoints and, therefore, a subdomain $\Omega <(0,{L}_{\mathrm{MB}})$ of the muscle. This correction factor can be determined based on the given fascicle coordinate input and does not need to be prescribed separately, for more information on the determination of ${f}_{c}$, see “Appendix 2.” Note, the operation of length scaling preserves the muscle’s height.

For scaling in the width direction, the muscle cross section was divided into two sides with individual scaling directions (cf. Fig. 2b). This distinction was made in order to preserve the muscle’s cross-sectional shape for muscles with non-planar aponeuroses. Fascicles on both sides of the dividing plane were shifted in a direction that considers the local aponeuroses directions on the respective side, which were once again obtained by principal component analysis (see “Appendix 3”). Magnitudes for the displacement in transversal direction $\Delta {y}_{\mathrm{F}}$ were obtained similar to Equation (2).

3 $$\Delta {y}_{\mathrm{F}}={y}_{\mathrm{F}}\cdot \frac{\Delta {W}_{\mathrm{MB}}}{{W}_{\mathrm{MB}}} .$$

Here, ${y}_{\mathrm{F}}$ represents the distance of the fascicle’s center-point from the dividing plane in $\mathbf{y}$ direction, where $\mathbf{y}$ can be ${\mathbf{y}}_{-}$ or ${\mathbf{y}}_{+}$ (cf. Fig. 2b).

In the next step, fascicles were lengthened by $\Delta {L}_{\mathrm{F}}$. In general, fascicles do not have to end on external aponeuroses. For compartmentalized muscles, they can also insert into internal aponeuroses, a previously reported finding for the rabbit PLA (Böl et al. 2015). To minimize fascicle protrusion of internal structures, fascicle endpoint displacement $\mathbf{u}$ was modeled in proportion to the point’s distance $d$ to the muscle’s line of action

4 $$\frac{\left|{\mathbf{u}}_{2}\right|}{\left|{\mathbf{u}}_{1}\right|}=\frac{{d}_{2}}{{d}_{1}},$$

where the index refers to the respective fascicle endpoint (cf. Fig. 2c), and $\left|{\mathbf{u}}_{1}\right|+\left|{\mathbf{u}}_{2}\right|=\Delta {L}_{\mathrm{F}}$. Note, this stretching in length direction leads to an increase in muscle height and to a reduction in fascicle curvature.

Finally, to capture changes in pennation angle $\beta$, the fascicles were rotated by $\Delta \beta$ in the vertical plane shown in Fig. 2d. Fascicles were rotated about their centers, resulting in a pure rotation without translation, since the center of the fascicles remained fixed during transformation. The transversal angle $\alpha$ remained unchanged. This operation again affects the muscle’s height.

**Fig. 3:**
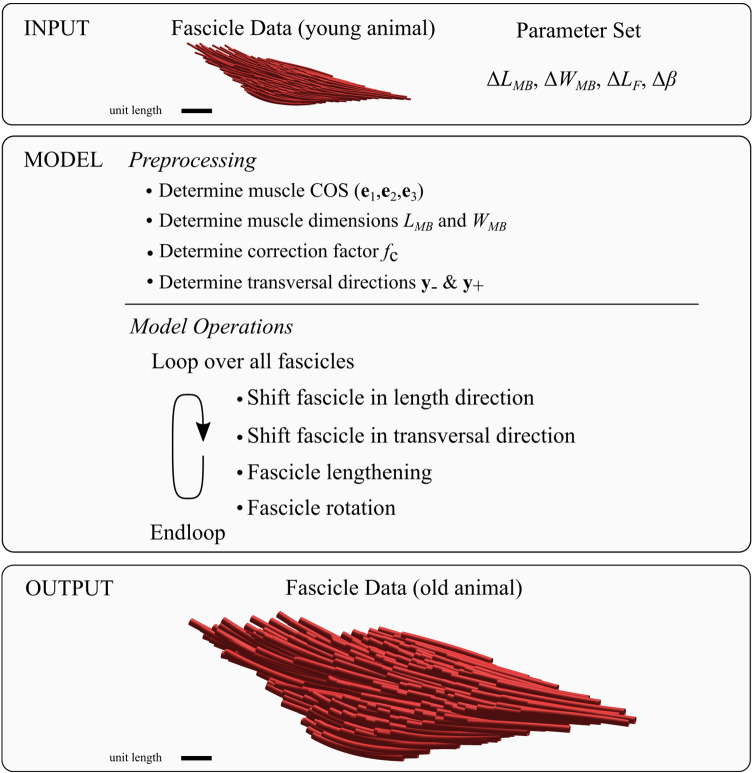
Visualization of the model’s workflow as well as its input and output. The input consists of a fascicle data set and the four input parameters muscle belly length change $\Delta {L}_{MB}$, muscle belly width change $\Delta {W}_{MB}$, fascicle length change $\Delta {L}_{F}$ and pennation angle change $\Delta \beta$. The model starts with a preprocessing step where the muscle’s coordinate system (${{\varvec{e}}}_{1},{{\varvec{e}}}_{2},{{\varvec{e}}}_{3}$) (cf. Fig. 2d), the muscle belly dimensions ${L}_{MB}$ and ${W}_{MB}$, the correction factor ${f}_{c}$ and the transversal directions ${{\varvec{y}}}_{-}$ and ${{\varvec{y}}}_{+}$ are determined (for further explanations see “Appendix 2” and “Appendix 3”). Subsequently, model operations are applied to each fascicle individually via a loop. The output resembles a new fascicle data set for the same muscle at a different size

The operations above are applied to each fascicle of the data set. Prescribed input arguments are the four parameters muscle belly length change $\Delta {L}_{\mathrm{MB}}$, muscle belly width change $\Delta {W}_{MB}$, fascicle length change $\Delta {L}_{\mathrm{F}}$ and pennation angle change $\Delta \beta$. Note that fascicle length change and pennation angle change are assumed constant and are, therefore, equal for every fascicle. Figure 3 shows a summary of the model, including its input and output. Since the above-mentioned architectural parameters were used as the input, they are not suitable for subsequent error estimation. Instead, we focused our analysis on the relative error between measured values and model predictions for the derived parameters aponeurosis length, aponeurosis width, muscle height, and muscle mass.

## Results

### Evolution of architectural parameters

**Fig. 4:**
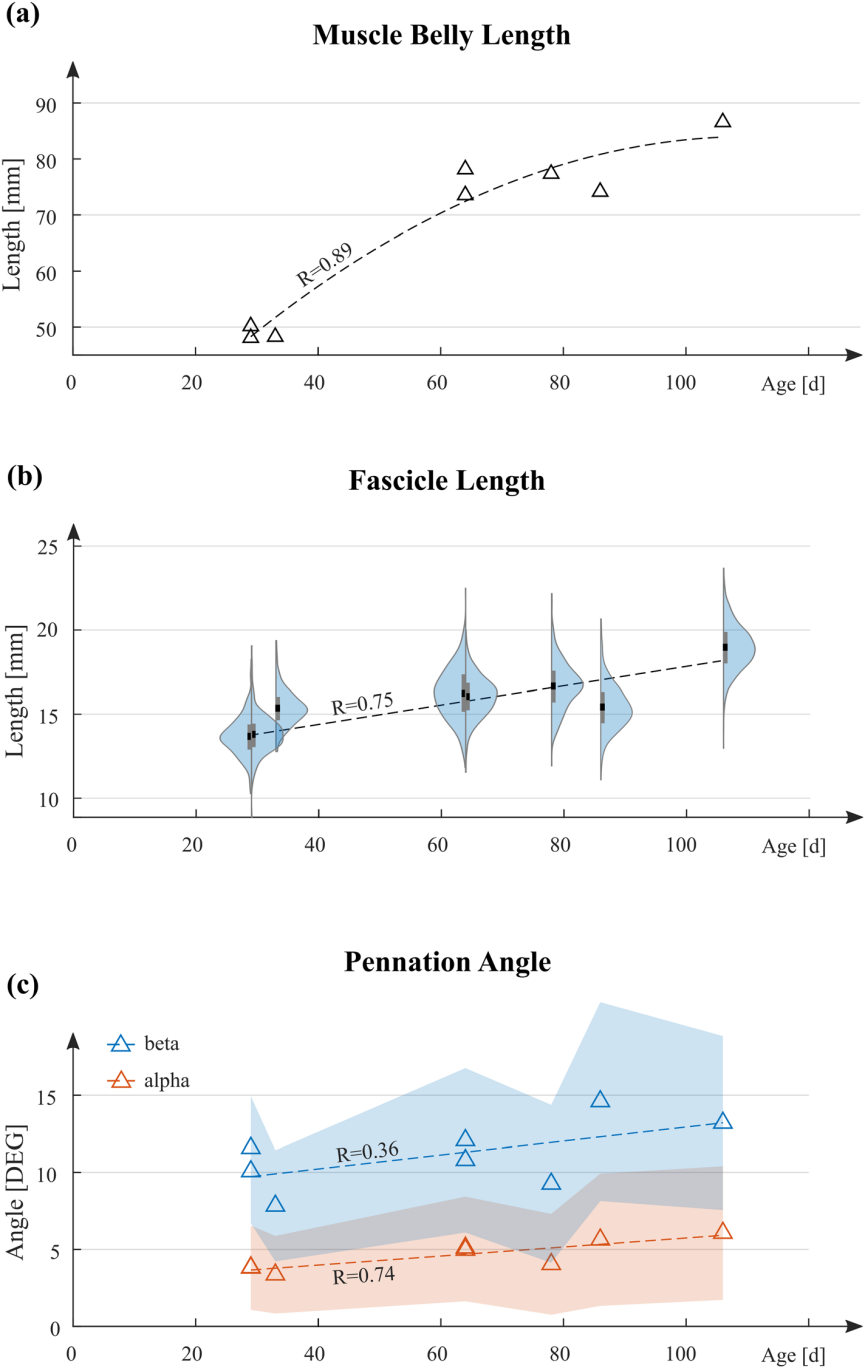
Evolution of the architectural parameters of the PLA. **a** Muscle belly length with respect to age. Triangles indicate measured lengths for individual muscles. The dashed line represents a quadratic fit through the dataset. **b** Violin plot for the fascicle lengths with respect to age. Areas indicate probability distributions. Bars show standard deviations, and dots represent mean fascicle lengths. The dashed line represents a linear regression line for the mean fascicle lengths of individual muscles. **c** The pennation angle with respect to age. Here, triangles represent measured mean angles. The dashed lines represent linear regression models, and the colored areas indicate standard deviations

During growth, all architectural parameters of the PLA experienced increases (cf. Fig. 4). From 29 to 106 d, muscle belly length increased by 73%, whereas mean fascicle length increased by 39% (p = 0.0037). The mean angle of pennation $\beta$ increased by 14% but this difference did not reach significance (p = 0.109). Moreover, muscle belly length growth seems to reach a saturation state (cf. Fig. 4a). Besides, fascicle length distributions for individual animals showed normal distribution patterns with similar standard deviations (cf. Fig. 4b, colored areas). The lateral angle $\alpha$ was notably smaller than $\beta$ (cf. Fig. 4c) and showed a strong correlation (R = 0.74) with age, however, absolute increases were small. For growth from 29 to 106 d, $\alpha$ and $\beta$ increased by 2.2° and 1.6°, respectively. Furthermore, both angles showed large standard deviations compared to the fascicle length.

**Fig.5:**
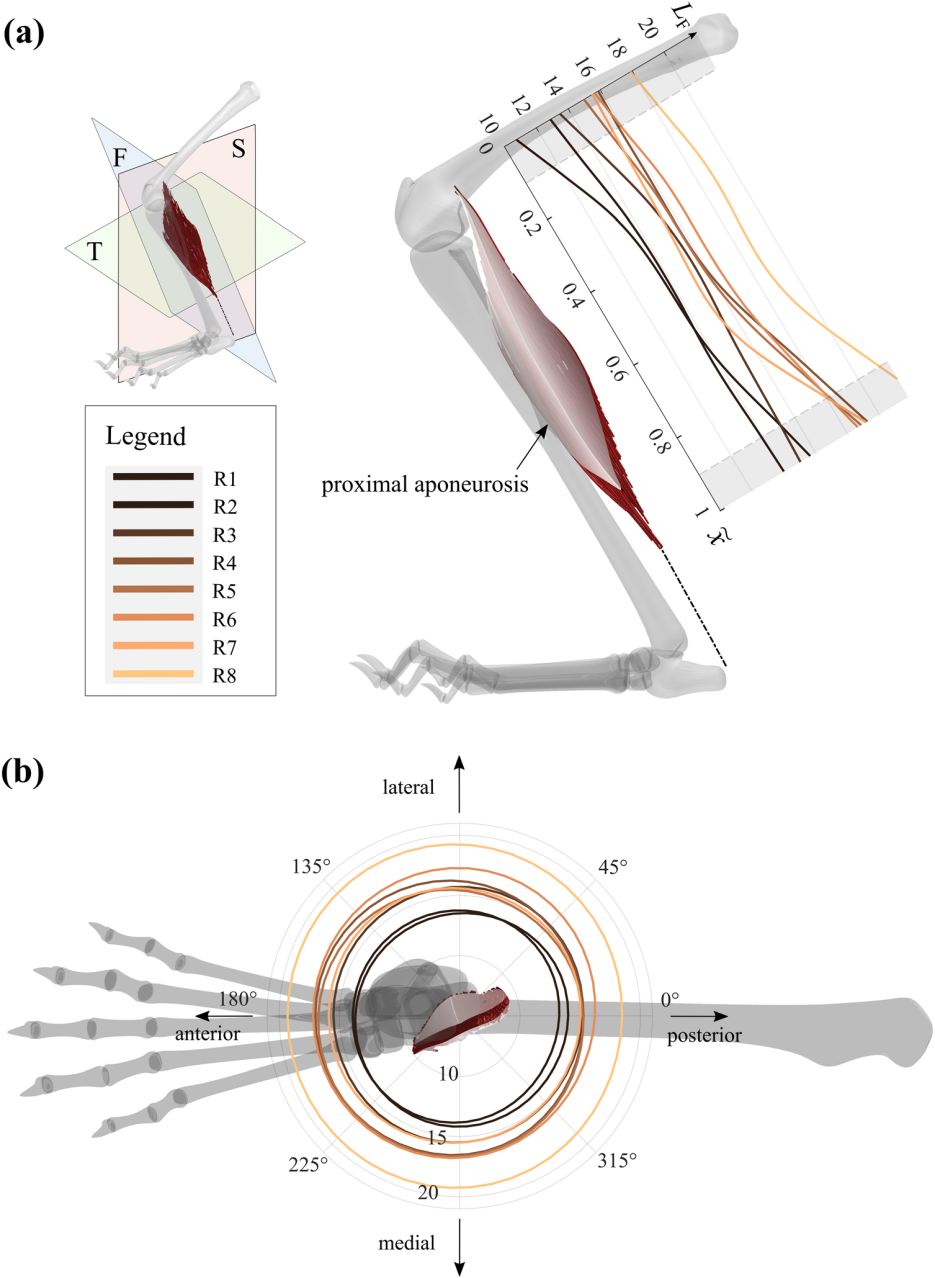
Growth in fascicle length for the transversal plane and muscle belly length axis. **a** Sagittal view on the leg with a plot of the fascicle length ${L}_{F}$ along the normalized muscle belly length axis $\stackrel{\sim }{{\varvec{x}}}$. **b** Fascicle length displayed in the transversal plane in polar coordinates. Data points were obtained by evaluating the polar angle for every fascicle in combination with its length. Lines represent smoothing spline fits through these data (MATLAB: smoothing parameter p = 0.999). Line colors refer to the same legend as in **a**

The fascicle length was further evaluated for the fascicle’s normalized midpoint position along the muscle belly length axis $\tilde{x }$ (cf. Fig. 5a). Results indicate increases in fascicle length from proximal to distal. However, there is no apparent change in the distribution pattern with age. Also, fascicle length was evaluated for the polar angle of its midpoint coordinates in the transversal plane (cf. Fig. 5b). Here, polar angles of 0° and 90° correspond to the posterior and lateral directions. Results show only a weak dependency of the fascicle length on the polar angle, where polar plots almost resemble circular shapes. Furthermore, there is no change in the distribution pattern from younger to older animals. For older animals, curves merely expand to higher lengths while maintaining their circular shapes.

Finally, we evaluated the aponeurosis length, aponeurosis width, PCSA, muscle mass, and free tendon length, which increased (from youngest to oldest animal) by 76%, 162%, 478%, 705%, and 81%, respectively. Detailed documentation of numerical data for architectural parameters can be found in Table 3.

### Model predictions

**Fig. 6:**
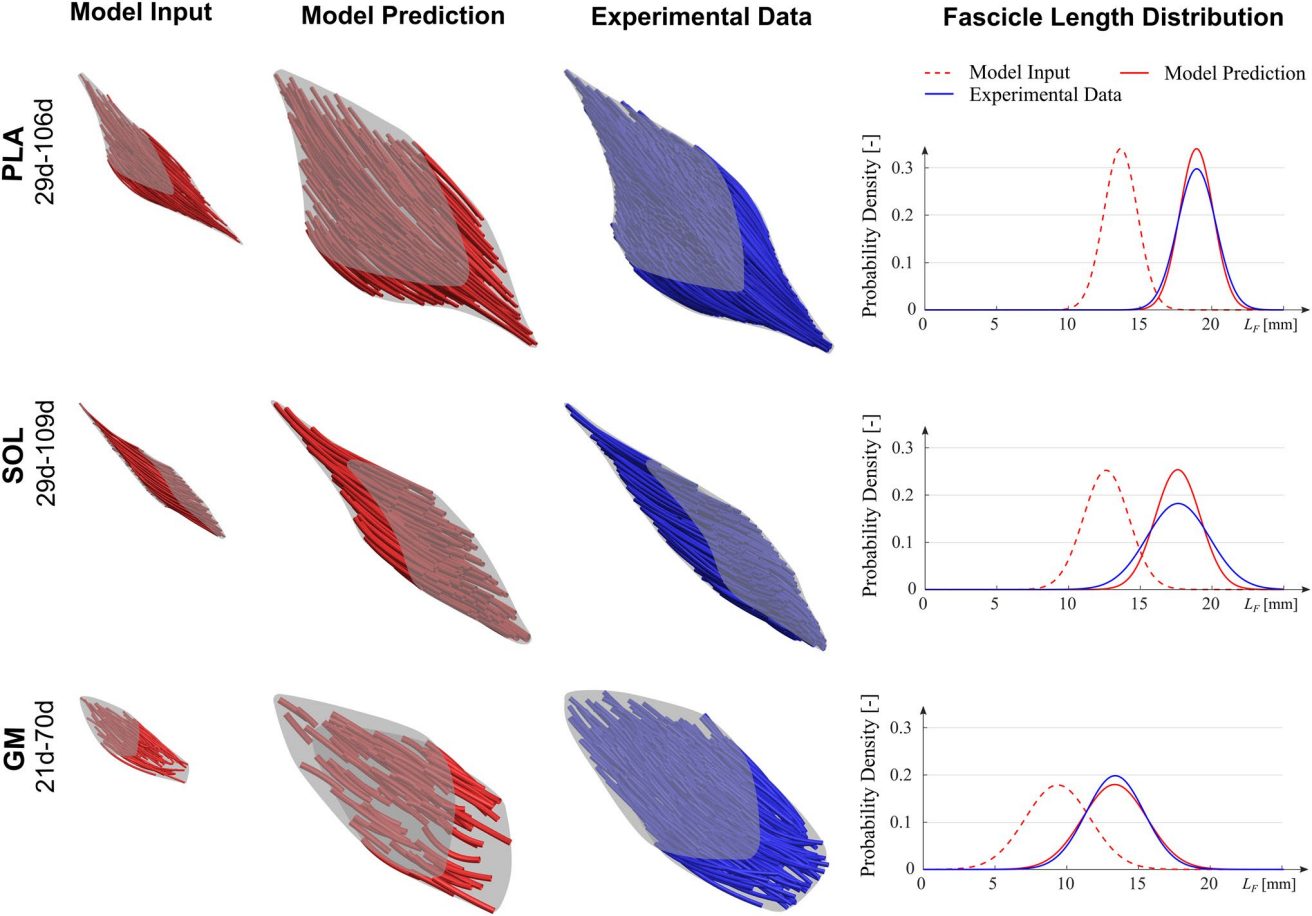
Model predictions and experimental data. (top row) Model prediction for the PLA of rabbit R8. Besides, model predictions for the muscles SOL (middle row) and GM (bottom row) are shown. Experimental data for the SOL and GM were taken from Papenkort et al. (2020) and Siebert et al. (2017), respectively. Note that the prediction's fascicle architecture appears sparser since the number of fascicles did not increase during model transformations. Especially for the youngest GM, only 78 fascicle traces were provided by the literature (Siebert et al. 2017), resulting in apparent gaps between the fascicles (bottom row, GM model prediction). For modeling purposes, the number of fascicles can easily be increased by interpolation. The aponeuroses were drawn based on fascicle endpoint locations. The right column shows the corresponding fascicle length distributions

**Fig. 7:**
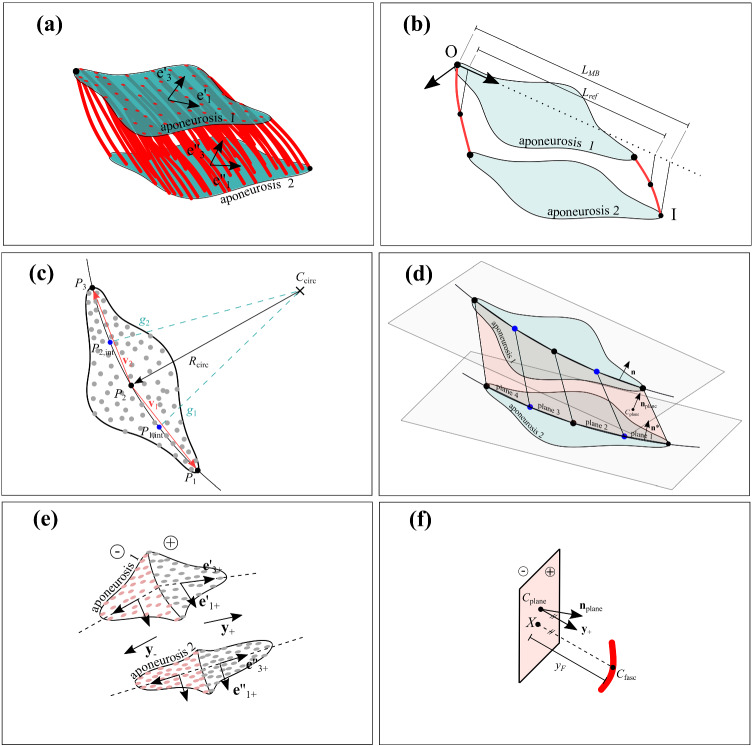
Length and transversal scaling. **a** Muscle belly with fascicles as red tubes. Fascicle endpoints correspond to points on an aponeurosis. ${{\varvec{e}}}_{1}^{{{\prime}}}$, ${{\varvec{e}}}_{3}^{{{\prime}}}$ and ${{\varvec{e}}}_{1}^{{{\prime\prime}}}$, ${{\varvec{e}}}_{3}^{{{\prime\prime}}}$ denote the first and second principal component of the aponeurosis points of the first and second aponeurosis, respectively. **b** Muscle belly with only its most proximal (O) and distal (I) fascicle. Here, ${L}_{MB}$ and ${L}_{ref}$ define the muscle belly length and the reference length for scaling, respectively. **c** Top view of a single aponeurosis. Individual aponeurosis points (fascicle endpoints) are drawn in gray. The aponeurosis is divided by a circle with the center-point ${C}_{circ}$ and the radius ${R}_{circ}$ that runs through the points ${P}_{1}$, ${P}_{1,int}$, ${P}_{2}$, ${P}_{2,int}$ and ${P}_{3}$. Its construction is based on the vectors ${{\varvec{v}}}_{1}$ and ${{\varvec{v}}}_{2}$ and the lines ${g}_{1}$ and ${g}_{2}$. **d** Dividing planes for the assignment of points to one side of the muscle. Individual planes are determined based on the aforementioned points ${P}_{1}$, ${P}_{1,int}$, ${P}_{2}$, ${P}_{2,int}$ and ${P}_{3}$ of each aponeurosis. The center-point ${C}_{plane}$ of the plane follows as the center-point of the four points defining the plane. The plane’s normal vector ${{\varvec{n}}}_{plane}$ is calculated based on the normal vectors ${\varvec{n}}$ and ${{\varvec{n}}}^{*}$ of line segments on aponeurosis 1 and aponeurosis 2. **e** Transversal directions of the muscle belly. Gray points indicate aponeurosis points assigned to the positive side of the muscle. Light red points indicate points assigned to the negative side of the muscle. Local directions ${{\varvec{e}}^{{\prime}}}_{1+}$, ${{\varvec{e}}^{{\prime}}}_{3+}$ and ${{\varvec{e}}}_{1+}^{{{\prime\prime}}}$, ${{\varvec{e}}}_{3+}^{{{\prime\prime}}}$ follow from principal component analysis of the respective aponeurosis and muscle side. The transversal directions ${{\varvec{y}}}_{+}$ is calculated based on ${{\varvec{e}}^{{\prime}}}_{3+}$ and ${{\varvec{e}}}_{3+}^{{{\prime\prime}}}$. **f** Transversal distance ${y}_{F}$ for a fascicle on the positive side of the muscle. ${y}_{F}$ can be computed based on the plane’s center-point ${C}_{plane}$, the plane’s normal vector ${{\varvec{n}}}_{plane}$, the positive transversal direction ${{\varvec{y}}}_{+}$ and the fascicle’s center-point ${C}_{fasc}$. $X$ denotes the projection of ${C}_{fasc}$ onto the dividing plane in direction of ${{\varvec{y}}}_{+}$

We investigated the model predictions for older animals (R3-R8) based on fascicle data from the youngest animal (R1). Note that R1 and R2 are the same age. The parameter input for each prediction could be obtained from Table 3. Figure 6 exemplarily shows the model prediction for the oldest animal (R8).

The relative error of the model for aponeurosis length, aponeurosis width, muscle height, and muscle mass is given in Table 1. Results show that errors stayed below ± 10% for the entire age range except for the muscle mass. Here, the maximum error reaches a value of −17.3%. On average, for all metrics and ages, the PLA's model error resulted in −0.9 ± 6.0%. The agreement of fascicle length distributions of experimental data and model predictions is shown in the right column of Fig. 6.

**Table 1:** Model errors for different rabbit calf muscles

Muscle	Age of prediction (d)	Aponeurosis length (%)	Aponeurosis width (%)	Muscle height (%)	Muscle mass (%)
PLA	33	5.3	3.8	− 5.9	0.4
PLA	64	− 2.1	0.4	8.5	− 3.6
PLA	78	− 4.8	0.4	2.2	4.7
PLA	86	− 4.4	0.5	− 7.2	− 17.3
PLA	106	− 2.5	1.2	8.1	− 6.5
**PLA Mean ± Std**	**–**	− **1.7 ± 4.1**	**1.3 ± 1.5**	**1.2 ± 7.4**	−**4.5 ± 8.3**
SOL	33	15.8	− 15.9	− 17.2	− 17.0
SOL	64	− 1.1	− 2.8	2.9	1.3
SOL	78	− 3.5	− 1.9	5.1	14.5
SOL	86	− 4.7	− 2.3	3.5	14.3
SOL	106	− 5.0	− 1.2	4.5	− 12.3
SOL	109	1.4	− 2.5	− 3.6	− 11.8
**SOL Mean ± Std**	**–**	**0.5 ± 7.9**	− **4.4 ± 5.6**	−**0.8 ± 8.6**	−**1.8 ± 14.0**
GM	37	13.8	10.5	− 12.7	− 0.1
GM	50	− 13.7	− 0.9	19.0	− 18.8
GM	70	− 1.7	0.3	14.8	− 3.8
GM	100	− 4.5	− 2.1	3.8	− 9.1
**GM Mean + Std**	**–**	− **1.5 ± 11.5**	**2.0 ± 5.8**	**6.2 ± 14.1**	− **8.0 ± 8.1**

For the PLA and SOL, the model input data were obtained from a 29 d old animal

For the GM, the initial data set was from a 21 d old animal. For the aponeuroses, values refer to the average length and width of the proximal and distal aponeurosis. Note that the prediction is shown for 5 PLA muscles, as R1 was the initial data, and there were two data sets for 29d and 64d (see Table 3). Experimental input and reference data for the SOL and GM were taken from (Papenkort et al. 2020) and (Siebert et al. 2017), respectively.

Next, the model was tested for different calf muscles. For this purpose, the SOL and GM were chosen. Fascicle data for these muscles were obtained from Papenkort et al. (2020) and Siebert et al. (2017), respectively. Results for 3D architectures and model errors are again shown in Fig. 6 and Table 1. For SOL, results are in a similar range compared to the results for the PLA. However, the muscle mass shows slightly higher errors, where individual errors are between 10 and 20%. The GM shows increased errors for the younger prediction ages, again not exceeding 20%, but very accurate results for older ages (cf. Table 1). Again, fascicle length distributions of model predictions and experimental data were in good agreement for both muscles (cf. Fig. 6 right column). The overall model error for all muscles, ages, and error metrics resulted in −1.0 ± 8.6%. It should be noted that the predictions of the GM were based on a comparatively sparse data set of 78 fascicles (Siebert et al. 2017), compared to 115 and 252 fascicles for the SOL and PLA, respectively.

## Discussion

In this study, an architectural model was developed that predicts the three-dimensional muscle architecture during maturation. The model was validated for three different calf muscles, namely PLA, SOL, and GM. An overall error of −1.0 ± 8.6% and a maximum error of 19.0% for all muscles (PLA, SOL, GM), error metrics (aponeurosis length, aponeurosis width, muscle height, muscle mass), and ages, indicated a good quality of the model’s predictions. We analyzed the PLA architecture of eight rabbits, ranging in age from 29 to 106 d, obtained by manual digitization for model development. This experimental method is considered to be very reliable since its data acquisition depends on the specimen's direct inspection. Results revealed major increases in muscle belly length (73%) that were accompanied by similar increases in aponeurosis length (76%) and even higher increases in aponeurosis width (162%). Fascicle length and pennation angle increased to a lesser extent by 39% and 14%, respectively.

### Model considerations

The developed four-parameter model incorporates changes in muscle belly length, muscle belly width, mean fascicle length, and mean pennation angle, and predicts the 3D fascicle architecture for an arbitrary age based on a given initial data set.

The scaling in muscle length, where fascicles are shifted in the aponeurosis length direction, was motivated by similar increases in muscle belly length (73%) and aponeurosis length (76%). However, the pronounced differences in aponeurosis length growth (76%) to aponeurosis width growth (162%) required independent scaling in the transverse direction. Furthermore, due to the non-planar geometry of the proximal, superficial PLA aponeurosis (cf. Fig. 5), with a distinct crest running centrally along the aponeurosis, the muscle cross section was divided into two parts, with separate directions (${\mathbf{y}}_{-}$, ${\mathbf{y}}_{+}$) for transversal scaling (cf. Fig. 2b). This distinction preserved the overall muscle shape during maturation, which was observed for the PLA. Changes in aponeurosis size are generally assumed to be connected to muscle fascicle thickness changes since aponeuroses represent the fascicles' attachment sites. Dominating contributions to muscle belly length growth by increases in fascicle width were also explicitly reported by Weide et al. (2015) for the human GM.

For the fascicle length, homogeneous growth was observed. Fascicle length distributions in Fig. 4b show a similar standard deviation for animals of different age. Moreover, Fig. 5 shows that distribution patterns for fascicle length are preserved during maturation. These findings motivated a constant lengthening of fascicles by $\Delta {L}_{\mathrm{F}}$.

Changes $\Delta \beta$ in pennation angle were comparably small but were nevertheless taken into account because of its connection to muscle height. Since muscle height changes were comparably small in absolute terms, incorporating even small pennation angle changes had a strong influence on the model results. Furthermore, more profound changes in the pennation angle, and muscle height, are documented for other muscles (Binzoni et al. 2001).

For the three muscles investigated in this study (PLA, SOL, GM), the model yielded accurate results with an overall error of −1.0 ± 8.6% and a maximum error of 19.0%. Due to the cross-sectional study design, experimental data for different ages are from different animals and include interindividual differences. To estimate those interindividual differences, animals of the same age were analyzed (R1 & R2 and R4 & R5). Results showed a maximum error of 18.0% (muscle mass) for that group, almost identical to the 19.0% found in model predictions. Individual errors for the other error metrics followed to 11.0% (aponeurosis length), 4.9% (aponeurosis width), and 12.1% (muscle height). Again, matching the model's variations, suggesting that model predictions lead to realistic architectural representations for a given age. Furthermore, the model enabled realistic predictions of fascicle length distributions for all muscles (cf. Fig. 6).

### Classification in the literature

To the best of the authors’ knowledge, there is only one study in the literature dealing with architectural changes in the developing rabbit PLA (Böl et al. 2016). However, this study did not measure 3D fascicle architecture but mainly focused on the PLA muscle-tendon complex's morphometric measures and its isolated aponeuroses. Linear regression models were evaluated so that data matched the growth period of 29 d to 106 d as investigated in the present study. A detailed comparison of the results is shown in Table 2.

**Table 2:** Development of selected architectural parameters during the growth period (29 to 106d) from Böl et al. (2016) and the present study

	Böl et al. (2016) % change │ abs. change	Present study % change │ abs. change
Aponeurosis length	106% | 72.0 mm	77% | 29.9 mm
Aponeurosis width	165% | 32.9 mm	163% | 12.5 mm
Muscle belly length	86% | –	73% | 36.4 mm
Muscle mass	544% | 7.94 g	703% | 6.13 g
PCSA	672% | 652 mm^2^	478% | 290 mm^2^
Fascicle length	4% | 0.5 mm	39% | 5.3 mm

Changes in architectural parameters are in general accordance with Böl et al. (2016). The deviations, e.g., in fascicle length growth, might be due to methodical differences. Böl et al. (2016) measured only a few fascicles from a specific muscle region for each muscle by caliper, which might have missed the overall growth trend apparent in this study, where the entire fascicle architecture was recorded. In another study on rabbits, Siebert et al. (2015) determined the pennation angle of adult rabbit PLA. For knee and ankle joint angles of 92° and 70°, respectively, they reported a mean pennation angle of 11.1 ± 4.8°, where the angle was measured as the spatial angle between the fascicle and the muscle’s line of action. Following that definition, our data yield an angle of 9.8 ± 4.5°, which agrees with the experimental data of Siebert et al. (2015). A similar pennation angle of 14.0 ± 1.2° was also found in adult mice (Roy and Edgerton 1995), at ankle and knee joint angles of approximately 90°. The increased angle might, in part, stem from increasing pennation angles at the shorter length.

### Functional aspects

The aforementioned large growth in muscle belly length is accompanied by a similar tendon length growth (cf. Table 3), leading to a change in the tendon-muscle fascicle length ratio (Siebert et al. 2017). This ratio is of critical functional importance (Biewener 1998; Mörl et al. 2016). Muscles with a small tendon-fascicle length ratio show only small amounts of elastic recoil and act primarily as motors in concentric contractions, as in the pigeon pectoralis muscle (Biewener 1998), the main motor for wing propulsion during the down-stroke phase. Conversely, muscles with a high tendon-fascicle length ratio act more like springs and can store and return large amounts of elastic energy. Prominent examples of such muscles are the calf muscles of terrestrial animals. Here, energy is periodically stored at the beginning of the stance phase and released at the end of the stance phase, initiating the swing phase. In the present study, values for the tendon-muscle fascicle length ratio increase from 1.06 to 1.38 for ages 29 to 106 days. These results support similar findings by Böl et al. (2016). The increased energy demand due to an increased body weight may make a more elastic, energy-conserving muscle more favorable. We want to emphasize that increases in aponeurosis lengths drive the increase in tendon–muscle fascicle length ratio. Aponeuroses exhibit complicated two-dimensional strain distributions under load that induce stiffness modulation (Azizi and Roberts 2009). During active contractions, positive strains in perpendicular direction account for a higher stiffness in the aponeurosis’s length direction (Raiteri 2018). The increased stiffness of the aponeurosis results in a more responsive system, which may be advantageous compared with a system in which the contribution of a tendon lacking the potential of stiffness modulation dominates the series elastic component length.

**Table 3:** Summary of the numerical data on the progression of architectural parameters with age

Rabbit ID	R1	R2	R3	R4	R5	R6	R7	R8	${\Delta }_{\%}$ R1 → R8
Age [d]	29	29	33	64	64	78	86	106	266
Animal mass [kg]	0.80	0.60	0.76	2.30	2.46	2.00	3.06	5.00	525
Ankle joint angle [°]	56.5	62.9	54.2	71.5	63.0	60.6	66.1	60.3	–
Knee joint angle [°]	81.6	83.4	82.6	88.4	87.8	89.8	83.7	87.1	–
Muscle belly length [mm]	50.8	50.4	49.1	78.6	74.2	77.5	74.0	87.2	72
Fascicle length [mm]	13.7 ± 1.2	13.8 ± 1.2	15.3 ± 1.0	16.2 ± 1.5	16.0 ± 1.2	16.6 ± 1.4	15.4 ± 1.4	19.0 ± 1.3	39
Pennation angle $\beta$ [°]	11.6 ± 4.6	10.1 ± 3.6	7.8 ± 3.6	10.8 ± 5.2	12.1 ± 5.4	9.3 ± 5.1	14.6 ± 6.4	13.2 ± 5.6	14
Muscle mass [g]	0.87	0.71	0.65	3.28	3.13	2.37	5.04	7.00	705
PCSA [mm^2^]	60.6	49.1	40.1	192.1	185.0	134.9	310.5	350.3	478
Free tendon length [mm]	14.5	14.2	13.6	22.6	22.4	7.6	30.5	26.3	81
Aponeurosis length [mm]	38.7	37.0	35.3	62.6	61.0	62.0	59.5	68.0	76
Aponeurosis width [mm]	7.7	7.3	6.9	14.6	14.6	12.4	17.6	20.2	162

The pennation angle refers to the angle $\upbeta$ in Fig. 2d. Parameters that refer to muscle fascicles refer to mean values for the entire muscle. The values for the aponeurosis length and width refer to mean values between the proximal and distal aponeurosis. An additional column at the right depicts the relative changes ${\Delta }_{\mathrm{\%}}$ between rabbit R1 and R8

Similar to the tendon length, growth in PCSA far exceeds the increases in fascicle length, resulting in a muscle with a higher fascicle width to length ratio. For thicker fascicles, an increased number of myofibrils in parallel leads to increased force production. Fascicle length, on the other hand, can directly be linked to the contraction velocity. Due to an increased number of sarcomeres in series, longer fascicles show a higher velocity of contraction. Comparably short muscle fascicles also lead to a shorter range of motion. An effect that may be partially be compensated for by architectural gearing (Azizi et al. 2008), in which muscle length changes exceed fascicle length changes during shortening contraction due to a change in the pennation angle, allowing the muscle to generate forces over a longer range of motion. During rabbit PLA development, our data show a clear trend in favor of force production with increasing age. Analysis of the aponeurosis width shows that increases in PCSA are primarily achieved by increases in muscle width. These increases in PCSA are presumably necessary due to an increasing overall body weight. However, a lower ratio of PCSA to muscle fascicle length, which favors contraction velocity, appears to be advantageous in predatory situations where younger animals are particularly vulnerable.

### Conclusion

In this study, the PLA architecture of rabbits during maturation was analyzed and an architectural model for 3D muscle growth was derived. Based on a specific, initial 3D data set, the model requires the input of only four parameters to predict the whole 3D fascicle architecture of the muscle at a different age. According to the model approach, growth could be described by a superposition of scaling in the length direction, scaling in the width direction, fascicle lengthening, and fascicle rotation, with all operations applied homogeneously over the entire domain.

The analysis of experimental data showed that muscle growth (72% in muscle belly length) is primarily facilitated by increases in aponeurosis dimensions (76% and 162% in length and width, respectively), whereas muscle fascicle length and pennation angle increased by only 39% and 14%, respectively. The model can make accurate predictions for the architecture of three different rabbit calf muscles, namely PLA, SOL, and GM, supporting the validity of the model assumptions and suggesting that it captures primarily unipennate muscles' general growth characteristics. The analysis of the error in this study focused on metrics related to the entire muscle, such as muscle height and volume, and yielded an overall error of − 1.0 ± 8.6%. Moreover, fascicle length distributions were accurately predicted by the model.

In the future, the presented model could be used in basic research and clinical applications to generate architectural datasets for unknown stages during growth that can be used as input for subsequent simulations on the mechanical behavior of skeletal muscle (Blemker et al. 2005; Seydewitz et al. 2019; Röhrle et al. 2017). To achieve realistic results in such simulations, it is essential to incorporate models that feature actual growth characteristics (Gans and Gaunt 1991; Woittiez et al. 1983) and avoid oversimplified approaches in which muscles are, for example, volumetrically scaled to a given target length.

## Data Availability

All necessary data and information are provided in the Online Resource so that published research is fully reproducible and the results reported can be verified.

## Appendix 1: animal information and results

See Table 3.

## Appendix 2: length scaling

For muscle belly length scaling, a fascicle is shifted in length direction, based on the location of its center-point ${x}_{F}$. In order to achieve muscle belly lengthening by $\Delta {L}_{MB}$, fascicle displacement $\Delta {x}_{F}$ can be calculated via

5 $$\Delta {x}_{F}={x}_{F}\cdot \frac{\Delta {L}_{MB}}{{L}_{ref}} ,$$

where ${L}_{ref}$ is the distance of the most proximal and most distal fascicle in muscle belly length direction, see Fig. 7b Comparing Eqs. (5) to (2), the correction term ${f}_{c}$ follows to

6 $${f}_{\mathrm{c}}=\frac{{L}_{MB}}{{L}_{ref}} .$$

## Appendix 3: transversal scaling

In order to preserve muscle shape during transversal scaling, the muscle belly is divided into two sides with individual scaling directions for fascicles on each side. The calculation of the muscle sides starts with determining the center line on an aponeurosis which is approximated by a circle (cf. Fig. 7c). To calculate the center line, first the most proximal and distal points (${P}_{1}$ and ${P}_{3}$) of the aponeurosis are determined. Furthermore, the center-point (${P}_{2}$) is calculated. Vectors from ${P}_{2}$ to ${P}_{1}$ (${{\varvec{v}}}_{1}$) and ${P}_{2}$ to ${P}_{3}$ (${{\varvec{v}}}_{2}$) represent secants of the circle. Therefore, a line ${g}_{1}$ perpendicular to ${{\varvec{v}}}_{1}$ that intersects ${{\varvec{v}}}_{1}$ half way, points in radial direction. In a similar way, we construct a line ${g}_{2}$. Since ${g}_{1}$ and ${g}_{2}$ both point in radial direction, they intersect at the center-point ${C}_{circ}$ of the circle. Now, the radius ${R}_{circ}$ of the circle can be identified based on the distance between ${C}_{circ}$ and ${P}_{2}$. We now want to approximate the circle in the region of interest by 4 straight line segments. Therefore, we determine two additional, intermediate points on the circle ${P}_{1,int}$ and ${P}_{2,int}$.

Based on the points [${P}_{1}$, ${P}_{1,int}$, ${P}_{2}$,${P}_{2,int}$,${P}_{3}$] of both aponeuroses, we defined four intersection planes via their normal form that divide the muscle into two sides (cf. Fig. 7d). Plane 1 for example was determined by [${P}_{1}$, ${P}_{1,int}$, ${P}_{1,int}^{*}$, ${P}_{1}^{*}$], where “*” indicates that the point belongs to the second aponeurosis. The center-point of these four points determines the point ${C}_{plane}$ of the plane. Its normal vector follows to

7 $${\varvec{n}}=({{\varvec{n}}}_{1}+{{\varvec{n}}}_{2})/|\left|{{\varvec{n}}}_{1}+{{\varvec{n}}}_{2}\right||,$$

where ${{\varvec{n}}}_{1}$ and ${{\varvec{n}}}_{2}$ are normal vectors of the line segments $({P}_{1},{P}_{1,int})$ and $({P}_{1}^{*},{P}_{1,int}^{*})$ in the respective aponeurosis plane.

For the transversal directions ${{\varvec{y}}}_{-}$ and ${{\varvec{y}}}_{+}$ we first assigned each fascicle endpoint on an aponeurosis to its respective side. Therefore, we determined the dividing plane with the closest center-point distance to the fascicle endpoint of interest. In the next step, we determined if the fascicle endpoint lied on the positive muscle side as indicated by the direction of the normal vector of the dividing plane, or on the negative, opposite side. Based on this analysis we carried out principal component analyses of the fascicle endpoints on each side of the two aponeuroses independently (cf. Fig. 7e). We defined the transversal direction for each side as.

8 $$\begin{aligned}{{\varvec{y}}}_{+}&=(\mathbf{e}^{{\prime}}_{3+}+{{\varvec{e}}}_{3+}^{{{\prime\prime}}})/||(\mathbf{e}^{\prime}_{3+}+{{\varvec{e}}}_{3+}^{{\prime \prime}})||,\nonumber\\ {{\varvec{y}}}_{-}&=(\mathbf{e}^{{\prime}}_{3-}+{{\varvec{e}}}_{3-}^{{{\prime\prime}}})/||(\mathbf{e}^{\prime}_{3-}+{{\varvec{e}}}_{3-}^{{\prime \prime}})||.\end{aligned}$$

For the transversal distance ${y}_{F}$ we first determined the dividing plane, closest to the fascicle of interest based on their respective center-points. Then, the distance ${y}_{F}$ followed as the length of a line segment that starts at the fascicle’s center-point and runs parallel to ${\varvec{y}}$ until it intersects with the dividing plane. Figure 7f exemplarily shows this line segment for a fascicle on the positive transversal side.

For computational purposes we can describe the plane in its normal form

9 $$\left({\varvec{x}}-{{\varvec{c}}}_{plane}\right)\cdot {{\varvec{n}}}_{plane} = 0,$$

where **x** is the location of an arbitrary point on the plane. For the line segment this point can be expressed as

10 $${\varvec{c}}_{fasc}-y_{F} {\varvec{y}}_{+}={\varvec{x}}.$$

Inserting Eq. (4) into Eq. (3) and solving for ${y}_{F}$ leads to

$${y}_{F} =({{\varvec{c}}}_{fasc}\cdot {{\varvec{n}}}_{plane}-{{\varvec{c}}}_{plane}\cdot {{\varvec{n}}}_{plane})/({{\varvec{y}}}_{+}\cdot {{\varvec{n}}}_{plane}).$$

## Code availability

An implementation of the architectural in MATLAB (MATLAB R2018b, The MathWorks, Inc., Natick, MA, USA) is provided in the online resource.
